# Annotations

**Published:** 1904-08-13

**Authors:** 


### ANNOTATIONS.

### The Curse of Juvenile Smoking.

We are glad to learn that the Bill for preventing juvenile smoking, which has just been introduced into the House of Commons, is seriously intended. It is not brought forward at the fag end of the session for show, but it embodies the deliberate views of Mr. Rigg and several members who think that the time has arrived when the question of legislation should be considered by all who have at heart the welfare of coming generations. The authors of the measure propose not only to prohibit smoking by persons under the age of 16, but also the sale of tobacco to persons under that age. The penalty, in the event of the Bill becoming law, would be 10s. to the smoker; 20s. for the first offence to the person supplying him, and 40s. for the second offence. No doubt objections will be raised as to the difficulties of administration, and on this point we suggest to Mr. Rigg that if he is not acquainted with the manner in which similar regulations are already enforced in the United States, it would be worth his while to pay a visit to America in order to obtain information first-hand. We are convinced, however, that, though misrepresentations as to age would be lightly made and as lightly accepted, the legislation adumbrated is perfectly practicable. The testimony as to the pernicious results of child-smoking is overwhelming, and the recommendation of the Commission on Physical Deterioration that means should be found to remedy the evil, merely emphasises the settled convictions of all who have given attention to the matter. No reasonable person denies that men often find moderate smoking beneficial as well as pleasant, and they should certainly be free to follow their inclination at discretion. But year by year the number of boys indulging in this habit, which is most damaging to their health, increases. The sooner the vice is put down by Act of Parliament the better, and we cannot conceive that, because it would interfere with the freedom of irresponsible lads to do themselves injury, because parents whose duty it is to bring up their sons properly may suffer in pocket, or because tobacconists object to undertake the obligations of citizens, there will be any formidable opposition.

### British Science Guild.

This new organisation, which has just been publicly launched, is an outcome of the address delivered last year by Sir Norman Lockyer in his capacity as President of the British Association. The proposal is to conduct a crusade throughout the Empire for the purpose of convincing both Governments and peoples that it is by the application of scientific methods to all branches of human endeavour that progress and the general welfare are to be attained. The Guild recognises that the scientific spirit, essential to all true progress, is often sadly lacking, even in some of those who are responsible for the control and direction of the nation's activities, and it aspires to remedy this evil by associating together all who are competent to advance its purposes. It may, indeed, be said that its motive is "efficiency"—a gospel which has also been forcibly preached from another platform. The Guild, however, not only proclaims an end to be gained, but suggests the method to be followed. In this we feel sure it will carry the very general sympathy of the medical profession. No class of men has more repeated opportunities for observing the indifference of the great body of the public to the practical value and significance of scientific truths; and equally none are more keenly aware of the failure of many well-meant social and philanthropic efforts conducted in complete ignorance of scientific method. If the Guild can guide public opinion to the need for recognising facts as a basis for organised action, and can place the truths of science in advance of sentiment and emotion, it will prove itself a valuable factor in the shaping of the national destiny. The task before it is a long and an arduous one, and success at the best must be gradual and remote. But if the campaign is efficiently conducted and is prosecuted with energy, the new Guild has a great future before it. We cordially wish it an abundant success.

### Women Inspectors.

There is no need nowadays to argue in favour of the policy of an attempt to secure for those engaged in workshops and factories protection against injurious influences resulting from the carelessness or selfishness of employers. And official inspection is especially required in factories where women are employed. Less expert in the capacity for co-operation and union than the stronger sex, women need as a consequence a greater measure of legal protection. Several authoritative statements which have recently gained publicity render it extremely doubtful whether this desirable end is being attained. Thus, it will surely cause some surprise to find that, according to a speech made in the House of Commons in the course of a debate on the Factory Acts, there are only eight inspectors responsible for the supervision of a million and a half of women workers. It is impossible to believe that so small a number of inspectors is adequate to secure the strict fulfilment of the provisions of the law. There is no need to frame a general indictment against the employer class, or to deny that many among them take an anxious and intelligent interest in the welfare of their workers. But it is necessary to recognise the existence of a minority of a different order, and to provide for these by efficient inspection. Confirmation of this view is afforded by a discussion of the subject at the Congress of the Sanitary Institute, where one of the women inspectors stated that defects in light supply, ventilation, and sanitary accommodation existed in greater or less measure in most workshops where women are employed. Every now and then, too, the public prints announce a conviction for some decided breach of the Factory Acts; and, in view of the small number of women inspectors, it is impossible to avoid a suspicion that others remain undetected. The Home Secretary has acknowledged the defect, and has undertaken to remedy it, at least in some degree. With this, in the meantime, it is necessary to be content, but we shall hope that the promise will be fully realised.

